# Cadmium resistance microbes and TiO_2_ nanoparticles alleviate cadmium toxicity in wheat

**DOI:** 10.1038/s41598-025-88371-z

**Published:** 2025-02-14

**Authors:** Muhammad Younas, Muhammad Nafees, Muhammad Munir, Sarah Owdah Alomrani, Muhammad Waseem, Mohammed Ali Alshehri, Pallab K. Sarker, Shafaqat Ali

**Affiliations:** 1 Department of Environmental Sciences, Government College University, Faisalabad, Pakistan; 2 State Key Laboratory of Pollution Control and Resource Reuse, School of the Environment, Nanjing University, Nanjing, 21023 Jiangsu China; 3 Date Palm Research Center of Excellence, King Faisal University, 31982 Al-Ahsa, Saudi Arabia; 4 Department of Biology, College of Science and Arts, Najran University, 66252 Najran, Saudi Arabia; 5 Department of Biology, Faculty of Science, University of Tabuk, 71491 Tabuk, Saudi Arabia; 6 Environmental Studies Department, University of California Santa Cruz, Santa Cruz, CA USA; 7 Department of Biological Sciences and Technology, China Medical University, Taichung, 40402 Taiwan

**Keywords:** Cadmium, Nanoparticles, *Staphylococcus aureus*, Wheat, Environmental sciences, Nanoparticles

## Abstract

Cadmium toxicity in the soil is an alarming issue, and among innumerable approaches, microbe-facilitated nanoparticle application for alleviation of Cd stress is a well-accepted technique. The present study explored the efficiency of combined TiO_2_-NPs and *Staphylococcus aureus M1* strains for Cd mitigation in wheat plants. Results depicted that Cd stress attenuates the growth attributes while the collective application of NPs and microbes significantly upsurges the growth attributes as contrasted to Cd treatment. Combined TiO_2_-NPs and microbes application increased the total chlorophyll (12), a (10), b (11), and carotenoids (13%) under Cd (50 mg kg^− 1^) compared to microbial treatment. MDA (4), H_2_O_2_ (3), and EL (5%) were significantly down-regulated with combined TiO_2_-NPs and microbes application under Cd (50 mg kg^− 1^) compared to microbial treatment. CAT (17), SOD (7), POD (8), and APX (29%) were increased with combined TiO_2_-NPs and microbes application under Cd (50 mg kg^− 1^) comparison to microbial treatment. Cd accumulation in roots (34), shoots (23), and grains (27%) were significantly reduced under Cd (50 mg kg^− 1^) with combined TiO_2_-NPs and microbes application, contrary to microbial treatment. Subsequently, combined TiO_2_-NPs and microbial strains *Staphylococcus aureus* M1 application is a sustainable solution to boost crop production under Cd stress.

## Introduction

Wheat is a commodity cultivated in Pakistan on around 8,069,000 hectares. The Pakistan Agricultural Research Council (PARC) estimates that the annual per capita consumption of wheat is 120Kg, ranking at the top among other crops in the world^1^. With a production capability of about 650 million tonnes annually, wheat is a staple of diets that contributes to more than 50% of the world’s population^2^. Wheat growth and morphology are severely disrupted under stress conditions, which has dangerous effects on plants. Heavy metal contamination has become a more significant problem due to quick urbanization and industrialization, persistent use of agrochemicals and fertilizer, excessive mining, and poor waste management in developing world like Pakistan; water is a significant problem for agriculture. Farmers now use industrial wastewater to satisfy their water needs^3^. These industrial wastewaters are highly contaminated with hazardous heavy metals, including Pb, Ni, Cd, Zn, Fe, and Mn. Because of their propensity to bioaccumulate in crops, heavy metals pose serious hazards to human health and the ecosystem. Crops grown under contaminated sites grow poorly and accumulate heavy metals through bioaccumulation. In the event of ingested exposure, these accumulated metals pose serious health risks to humans and livestock^4^. The uptake of toxic elements from contaminated soil by plants can trigger an overproduction of reactive oxygen species (ROS), leading to impaired physiological functions and stunted plant growth accumulation of toxic elements by plants from polluted soil can induce the excessive formation of reactive oxygen species (ROS), thereby causing retarded plants’ physiological attributes^5^. The contamination of soil with lead (Pb) and cadmium (Cd) has become a pressing concern among researchers due to the detrimental impact of these toxic metals on food safety, human health, and ecosystem well-being^6^.

Cadmium (Cd) is one of the most potent heavy metals in contaminated soil. Cd is a versatile element that is easily absorbed by prokaryotic plant cells. Heavy metals cause food contamination, which is why these metals are the source of significant environmental, economic, and social issues worldwide, with grave consequences for human health^7^. Cd poisoning has been proven to produce several physiological anomalies, such as decreased respiration rate, photosynthesize, antioxidative enzymes, and reactive oxygen species. The sedentary nature of plants has driven the development of a sophisticated, multifaceted antioxidant defense mechanism. This complex system comprises a multitude of enzymatic components, including superoxide dismutase (SOD), catalase (CAT), peroxidase (POX), glutathione peroxidase (GPX), glutathione reductase (GR), glutathione S-transferases (GST), ascorbate peroxidase (APX), and monodehydroascorbate reductase. These enzymes play a vital role in enabling plants to withstand and adapt to various environmental stresses^8^. The impact of Cd on plant development and metabolic parameters of wheat transfected the breeding lines ranging in cadmium resistance^9^. Therefore, Cd polluted agricultural soil has become a global environmental problem that restricts the sustainable development of human society and needs to be solved urgently.

Phytoremediation, the strategic deployment of plants to eliminate environmental pollutants, offers a multifaceted solution that surpasses conventional remediation methods. This innovative approach boasts simplicity, efficiency, and cost-effectiveness while minimizing the ecological footprint. By enabling on-site remediation, phytoremediation streamlines logistics, reduces human and wildlife exposure, and promotes a more sustainable and environmentally conscious cleanup process^10^. Phytoremediation is one of the most suggested environmentally friendly and cost-effective remediation methods for heavy metals in soil^11^. Plant evolution-boosting rhizobacteria is a beneficial microbial population in the rhizosphere that can improve soil health and plant production while also changing the absorptive characteristics of plant roots in contaminated soils^12^. Microorganisms have various factors that limit the bioavailability or toxicity of metals and metallics through biomethylation and transformation, known as heavy metals resistant microbes, to solve the harmful effects of metals and metallics. The growth-promoting impact of PGPR on wheat growth in heavy metal-contaminated soil was discovered. Furthermore, PGPR may improve plant phytoremediation ability in Cd-contaminated soil^13^.

Titanium is the 9th most significant prevalent component in the earths crust and the second most rich transition metal^14^. Many Nanomaterials, such as titanium dioxide nanoparticles (TiO_2_ NPs), were first utilized to boost the production of edible plants^15^. Engineered nanomaterials (ENMs) have several distinguishing characteristics, including surface area, chemical composition, surface reactivity, charge, form, and media interactions. These physical, chemical, thermal, magnetic, optical, and biological qualities have resulted in various products containing food and everyday household items^16^. Titanium dioxide nanoparticles have been launched to expand seed germination in plants and broaden plant bodies’ growth parameters^17^. The integration of nano-zero valent iron (nZVI) with eggshell biochar (ESB) and activated carbon (AC) significantly enhanced the immobilization of lead (Pb) and cadmium (Cd) in soil, surpassing the efficacy of ordinary eggshell biochar. Consequently, this innovative treatment reduced the bioaccumulation of Pb and Cd in the edible parts of *Brassica chinensis* L. by a substantial margin. Furthermore, the nZVI-ESB/AC treatment exhibited remarkable benefits, including augmented plant growth, diminished oxidative stress indicators (by 1.5-2 folds), and a notable increase in the relative abundance of beneficial Bacilli and Clostridia bacteria (by 52–67% and 10–15%, respectively. Incorporating iron in nZVI-ESB/AC significantly boosted the activity of antioxidant enzymes, thereby mitigating oxidative stress in plants. This synergistic effect substantially reduced the production of reactive oxygen species (ROS) and lipid peroxidation, ultimately enhancing plant tolerance to heavy metal stress^18^.

The findings demonstrated that nano-zero valent iron (nZVI)-modified eggshell biochar effectively mitigated the bioavailability of lead (Pb) and cadmium (Cd) in soil, achieving reductions of 69–75% and 62–65%, respectively. Moreover, this innovative treatment significantly decreased the leachability and toxicity of Pb and Cd by 53–66% and 68–75%, respectively. Consequently, the risk of carcinogenic exposure and hazard index for children and adults were substantially lowered, with reductions of 35–47% for Pb and 25–36% for Cd^19^.

Numerous research studies have shown that cadmium-resistant microbes like *Staphylococcus aureus* and TiO_2_ NPs can decrease Cd poisonousness in wheat by enhancing wheat growth and decreasing uptake. Minimal data was found regarding the combined application of *Staphylococcus aureus* strains and TiO_2_ NPs to alleviate Cd stress. Henceforth, the present research exhibited the effects of alone and collective implications of microbial strains and TiO_2_ NPs on wheat plants. A knowledge gap exists in the current literature regarding the synergistic effects of combining Staphylococcus aureus M1 strains with TiO_2_ nanoparticles for mitigation purposes. This study aims to bridge this gap by investigating microbes and nanoparticles’ individual and combined impacts. The specific aims of the current study were (i) to explore the effect of mutual application of *Staphylococcus aureus* M1 strains and TiO_2_ NPs on growth and yield characteristics, (ii) to scrutinize the influence of combined application on oxidant and antioxidant enzyme activities, and (iii) examine the impact of alone and combined application on Cd uptake and accumulation.

## Materials and procedures

### Soil evaluation

The soil was arranged from the Ayub agriculture research field for the experiment and put through a 2 mm sieve. Typical methods were employed for the primary classification of the soil, including particle size. An exploration in laser diffraction soil particle size distribution analysis to achieve intimate results with the sieve and pipette method. Electrical conductivity (EC) and pH of the soil extract were calculated by soil-to-water ratio 1:25 through horizontal shaking for two hours. The metals concentration in the soil was investigated with the typical practice^20^. The granularity of soil organic matter was estimated using the Walkley-Black technique^21^. Calcium carbonate was projected using the calcimeter approach^22^. The complete physicochemical characteristics of soil are presented in (Table 1).

**Table 1:** Analysis of Soil used for this experiment.

Soil	Units
Textural Class	Sandy Clay Loam
Sand	61.2%
Silt	14%
Clay	24.8%
pH	7.51
EC	1.03 dS m^− 1^
HCO_3_ ^− 1^	2.99 mmol L^− 1^
Total nitrogen	0.05%
Available P	2.17 mg kg^− 1^
K^+^	0.09 mmol L^− 1^
Cl^− 1^	4.78 mmol L^− 1^
Ca^+ 2^ + Mg^+ 2^	13.76 mmol L^− 1^
Available Cd	0.08 mg kg^− 1^

### Seeds immunization with Cd-tolerant strains Staphylococcus aureus M1

Wheat cultivar Lasani 2016 was used in our experiment, and seeds were collected from Ayyub Agricultural Research Institute Faisalabad, Pakistan, with permission. A specific bacterial strain was grown in 250mL of nutrient broth media and cultured for 24 h at 37 °C on a rotary shaker set at 150 rpm to manufacture bacterial infusion^23^. The culture media was obtained for the night using a centrifuge set to 10,000 rpm for ten minutes and removing the supernatant. As shown in^23^, the attained bacterial pallet was gathered, disinfected with double distilled water that had been cleaned, and suspended in a regular salty mixture (0.85% NaCl). The seeds were superficially disinfected by submerging them in hydrogen peroxide (10% H_2_O_2_) for 30 min following the inoculum preparation^23^. The bacterial inoculum was employed on these seeds by placing the seeds in a binary capacity of bacterial suspension on a rotary shaker set to 37 °C for two hours at 90 rpm. Carboxymethyl cellulose (2%) supported the inoculum add-on seeds. These seeds are inoculated. These seeds were placed in a 1:1 w/w mixture of clay and peat moss, well mixed to provide a suitable covering, and incubated for 12 h in the dark^23^. Untreated seeds were sown in the control treatment.

### Pot experiment

Seeds were sown in pots to maintain ordinary environmental circumstances (temperature 28/20°C day/night, relative humidity 67 ± 5%) in the Botanical Garden of Government College University, Faisalabad, Pakistan. Sowing was done in plastic pots in sieved soil. Every pot was filled with 5 kg of soil with a specific experiment scheme. According to the treatment plan, Cd was used with different concentrations (50 and 100 mg kg^− 1^). To ensure reliability and accuracy, the experiment was designed with triplicates for each treatment and three replicates for the control group and all other treatments, thereby allowing for robust statistical analysis and validation of the results. The experimental design employed was a Completely Randomized Design (CRD), where treatments were randomly assigned to experimental units to minimize bias and ensure unbiased estimation of treatment effects. Five seeds were applied to every pot to germinate the plant. When germination started, the thinning was done to level the number of plants in every pot 3 plants were left in each pot. Fertilizer was applied, which were salt of di ammonium and potassium sulfate in the form of N, P, and K with a ratio of 110:45:1.8 kg ha^− 1^_,_ respectively. After 15 days of germination foliar spray of TiO_2_ nanoparticles (25, 50 ppm L^− 1^) was applied, and there were no Cd, NPs, and microbes in the control.

### Plants harvesting

After four months of sowing, plants were harvested. After harvesting, plants were classified into different parts of plants, e.g., roots, shoots, spikes, and grains, and plant height, weight, and spike length were noted. HCl (0.1%) acid was used to wash the roots of plants to eliminate roots’ surface metals and cleaned out with double distilled water to get the dry weight of roots and shoots. The oven was used for 72 h at 70 °C, and the roots and shoots were weighted and then crushed into tiny parts for others’ scrutiny.

### Growth, yield, and photosynthetic pigments

First of all, the growth attributes like root and shoot length (cm), weight (g) fresh and dry, spike length (cm), number of tillers, and grain weight (g) were estimated. Chlorophyll parameters such as (chlorophyll a, b, total chlorophyll, and carotenoid contents) were measured. For this purpose, 0.5 g of fresh leaves were submerged in 80% acetone nightly at -4 °C, and then the obtained extract was centrifuged with a spectrophotometer at the proposed wavelengths^24^.

### Evaluation of MDA, EL, H2O2, and antioxidant enzyme activities

Thiobarbituric acid (0.1%) was used to estimate the malondialdehyde (MDA) parameter, as per^25^ and Abbas et al.^26^ protocols. The Dionisio and Tobita^27^, approach was used for the EL approximation. Two stages made up the removal process: first, samples were separated at 32 °C for two hours to determine the solutions early EC, and then the method was repetitive at 121 °C for twenty minutes to gain the solution’s concluding EC. The internal mechanisms of H_2_O_2_ were demonstrated using the methodology proposed by Jana and Choudhuri^28^. After centrifugation, these samples were mixed with 50 milligrams of pH 6.5 phosphate buffer and centrifuged for 20 min. Following centrifugation, 20% vol/vol H_2_SO_4_ was inserted into the ultra-spin extract and centrifuged for 15 min. At 410 nm, the optical density was measured.

Phosphate buffer (0.5 M at pH 7.8) was mixed with samples to analyze the activity of peroxidase (POD) and superoxide dismutase (SOD)^29^. The ascorbate peroxidase (APX) activity was calculated, while the CAT activity was estimated using the methodology of Aebi et al.^30^.

### Evaluation of metal accumulation

The availability of Cd concentrations was assessed in triplicate samples of roots, shoots, and grain. For this purpose, diacid HNO_3_: HClO_4_ (3:1 v/v) digestion was done. Following digestion, the atomic absorption spectrophotometer (AAS) was used to measure the amount of Cd in the samples to identify metal concentration.

### Data examination

After collecting all data, investigations were conducted using SPSS Statistics software Version 21, and analysis of variation (ANOVA) at 5% probability was carried out. Tukey’s HSD post hoc test was completed for multiple evaluations of triplicates.

## Results

### Growth and yield characteristics

**Fig. 1:**
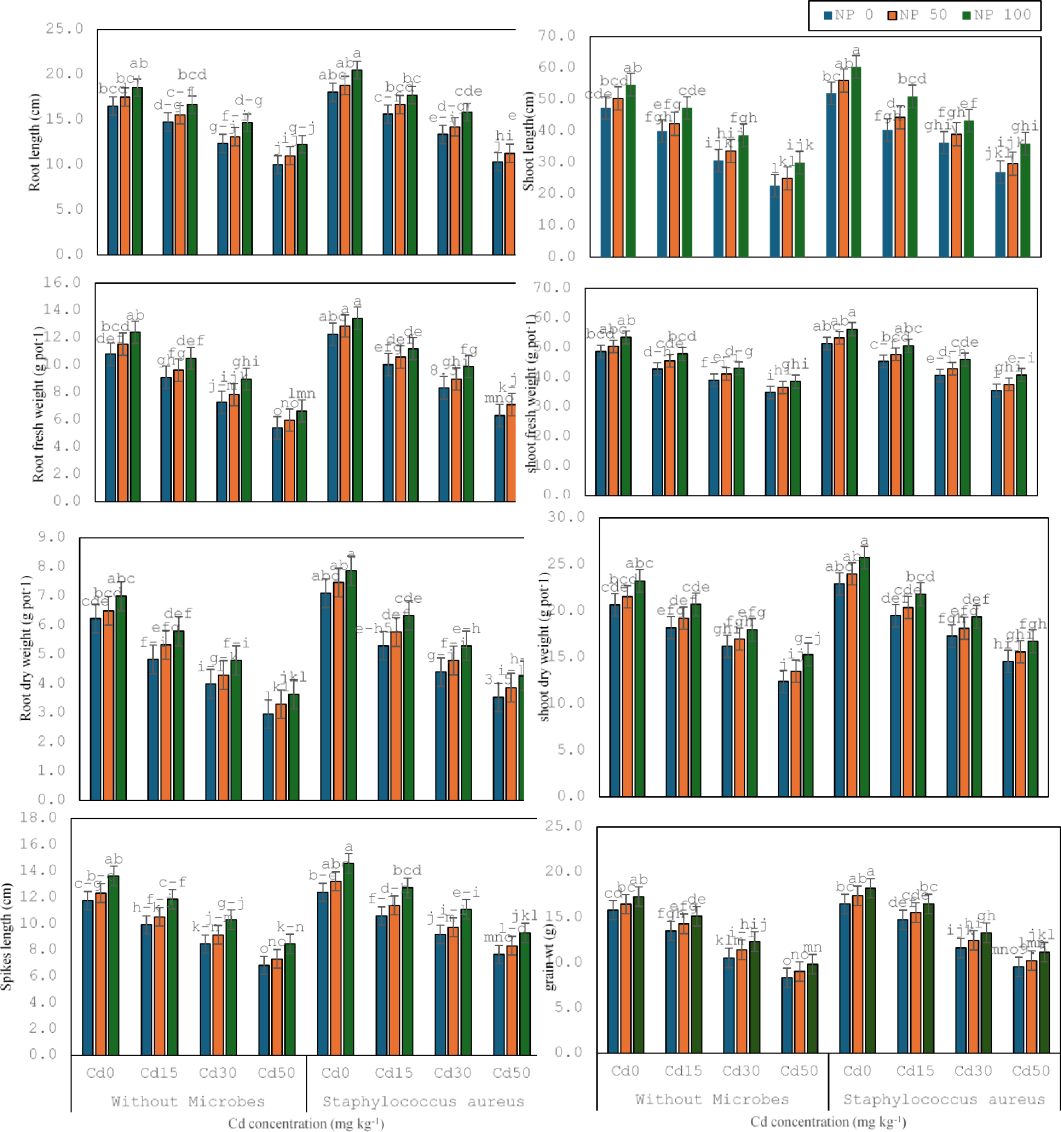
Effect of microbes on root length, shoot length, root fresh weight, shoot fresh weight, root dry weight, shoot dry weight spike length and no of leaves of wheat plant under the cadmium (0, 15, 30 and 50 mg kg^− 1^). Showing standard deviation at *p* ≤ 0.05 level with mean of three replications.

The existing study was arranged to estimate the bio-toxic consequences of cadmium on several structural, physical, and biochemical constraints of wheat and to evaluate the reducing ability of cadmium opposing bacteria (*S. aureus* M1) in synergistic relationships with TiO_2_ NPs (Fig. 1). It was noticed that Cd 15 mg kg^− 1^ decreased the shoot and root length (15, 10%), shoot and root fresh weight (12, 15%), shoot and root dry weight (11, 22%), spike length (16%) and No. of leaves (30%) and grain weight (as compared to control. It was noticed that Cd 30 mg kg^− 1^ decreased the shoot and root length (23, 20%), shoot and root fresh weight (9, 20%), shoot and root dry weight (11, 36%), spike length (15%), No. of leaves (17%) and grain weight (22%), as contrasted to Cd 15 mg kg^− 1^. It was noticed that Cd 50 mg kg^− 1^ declined the shoot and root length (26, 19%), shoot and root fresh weight (11, 26%), shoot and root dry weight (23, 52%), spike length (19%), No. of leaves (20%) and grain weight (21%), compared with Cd 30 mg kg^− 1^.

*S. aureus* M1 inoculation enhanced the shoot and root length (10, 9%), shoot and root fresh weight (6, 14%), shoot and root dry weight (11, 14%), spike length (5%), No. of leaves (12%) and grain weight (4%) under no stress, compared without inoculation. Whereas, with *S. aureus* M1 inoculation enhanced the shoot and root length (1, 7%), shoot and root fresh weight (6, 10%), shoot and root dry weight (7, 10%), spike length (100%), No. of leaves (44%) and grain weight (9%) under Cd 15 mg kg^− 1^, rivalled without inoculation. *S. aureus* M1 inoculation enhanced the shoot and root length (18, 9%), shoot and root fresh weight (4, 15%), shoot and root dry weight (7, 10%), spike length (9%), No. of leaves (47%) and grain weight (11%) under Cd 30 mg kg^− 1^, contrary to without inoculation. Similarly, with *S. aureus* M1 inoculation enhance the shoot and root length (12, 3%), shoot and root fresh weight (2, 17%), shoot and root dry weight (17, 19%), spike length (12%), No. of leaves (33%) and grain weight (14%) under Cd 50 mg kg^− 1^, equated without inoculation.

TiO_2_ 50mg L^− 1^ NPs enhanced the shoot and root length (6, 6%), shoot and root fresh weight (3, 7%), shoot and root dry weight (4, 4%), spike length (5%), No. of leaves (11%) and no of grains (7%) under no toxicity, parallel to control without TiO_2_ 50mg L^− 1^ NPs. Whereas TiO_2_ 50mg L^− 1^ NPs enhanced the shoot and root length (6, 5%), shoot and root fresh weight (6, 6%), shoot and root dry weight (6, 10%), spike length (6%), No. of leaves (6%) and no of grains (6%) under Cd 15 mg kg^− 1^, associated without TiO_2_ 50mg L^− 1^ NPs. TiO_2_ 50mg L^− 1^ NPs enhanced the shoot and root length (10, 6%), shoot and root fresh weight (5, 8%), shoot and root dry weight (5, 7%), spike length (8%), No. of leaves (7%) and grain weight (9%) under Cd 30 mg kg^− 1^, associated without TiO_2_ 50mg L^− 1^ NPs. TiO_2_ 50mg L^− 1^ NPs enhanced the shoot and root length (10, 10%), shoot and root fresh weight (5, 10%), shoot and root dry weight (9, 11%), spike length (7%), No. of leaves (8%) and grain weight (8%) under Cd 50 mg kg^− 1^, related to without TiO_2_ 50mg L^− 1^ NPs. Similarly, TiO_2_ 100mg L^− 1^ NPs enhanced the shoot and root length (9, 12%), shoot and root fresh weight (10, 15%), shoot and root dry weight (12, 12%), spike length (16%), and No. of leaves (11%) and grain weight (9%) under no stress, compared to TiO_2_ 50mg L^− 1^ NPs. TiO_2_ 100mg L^− 1^ NPs enhanced the shoot and root length (11, 13%), shoot and root fresh weight (5, 7%), shoot and root dry weight (8, 9%), spike length (13%), No. of leaves (6%) and grain weight (6%) under Cd 15 mg kg^− 1^, contrasted to TiO_2_ 50mg L^− 1^ NPs. TiO_2_ 100mg L^− 1^ NPs enhanced the shoot and root length (15, 15%), shoot and root fresh weight (4, 14%), shoot and root dry weight (6, 8%), spike length (12%), No. of leaves (7%) and grain weight (9%) under Cd 30 mg kg^− 1^, associated with TiO_2_ 50mg L^− 1^ NPs. Correspondingly, TiO_2_ 100mg L^− 1^ NPs enhanced the shoot and root length (20, 11%), shoot and root fresh weight (11, 10%), shoot and root dry weight (13, 10%), spike length (15%), No. of leaves (7%) and grain weight (9%) under Cd 50 mg kg^− 1^, related to TiO_2_ 50mg L^− 1^ NPs.

Co-application of TiO_2_ 50mg L^− 1^ NPs and *S. aureus* M1 inoculation enhanced the shoot and root length (10, 9%), shoot and root fresh weight (6, 13%), shoot and root dry weight (7, 10%), spike length (9%), No. of leaves (19%) and grain weight (5%) under no stress, aligned with respective treatment without TiO_2_ 50mg L^− 1^ NPs. The co-application of TiO_2_ 50mg L^− 1^ NPs and *S. aureus* M1 inoculation enhanced the shoot and root length (10, 7%), shoot and root fresh weight (5, 6%), shoot and root dry weight (4, 9%), spike length (8%), No. of leaves (8%) and grain weight (5%) under Cd 15 mg kg^− 1^, parallel with corresponding treatment without TiO_2_ 50mg L^− 1^ NPs. The co-application of TiO_2_ 50mg L^− 1^ NPs and *S. aureus* M1 inoculation enhanced the shoot and root length (7, 6%), shoot and root fresh weight (6, 8%), shoot and root dry weight (5, 9%), spike length (6%), No. of leaves (5%) and grain weight (7%) under Cd 30 mg kg^− 1^, associated with corresponding treatment without TiO_2_ 50mg L^− 1^ NPs. The co-application of TiO_2_ 50mg L^− 1^ NPs and *S. aureus* M1 inoculation enhanced the shoot and root length (19, 2%), shoot and root fresh weight (3, 19%), shoot and root dry weight (16, 17%), spike length (14%), No. of leaves (46%) and grain weight (13%) under Cd 50 mg kg^− 1^, contrasted with respective treatment without TiO_2_ 100mg L^− 1^ NPs. Co-application of TiO_2_ 100mg L^− 1^ NPs and *S. aureus* M1 inoculation enhanced the shoot and root length (8, 9%), shoot and root fresh weight (7, 4%), shoot and root dry weight (6, 5%), spike length (8%), No. of leaves (6%) and grain weight (5%) under no stress, contrasted with corresponding treatment without TiO_2_ 100mg L^− 1^ NPs. The co-application of TiO_2_ 100mg L^− 1^ NPs and *S. aureus* M1 inoculation enhanced the shoot and root length (15, 6%), shoot and root fresh weight (6, 6%), shoot and root dry weight (7, 10%), spike length (12%), No. of leaves (4%) and grain weight (6%) under Cd 15 mg kg^− 1^, linked with corresponding treatment without TiO_2_ 100mg L^− 1^ NPs. The co-application of TiO_2_ 100mg L^− 1^ NPs and *S. aureus* M1 inoculation enhanced the shoot and root length (11, 12%), shoot and root fresh weight (7, 10%), shoot and root dry weight (7, 10%), spike length (14%), No. of leaves (9%) and grain weight (8%) under Cd 30 mg kg^− 1^, compared with corresponding treatment without TiO_2_ 100mg L^− 1^ NPs. The co-application of TiO_2_ 10mg L^− 1^ NPs and *S. aureus* M1 inoculation enhanced the shoot and root length (21, 11%), shoot and root fresh weight (7, 8%), shoot and root dry weight (7, 10%), spike length (11%), No. of leaves (5%) and grain weight (9%) under Cd 50 mg kg^− 1^, equated with corresponding treatment without TiO_2_ 100mg L^− 1^ NPs.

### Photosynthetic pigments

**Fig. 2:**
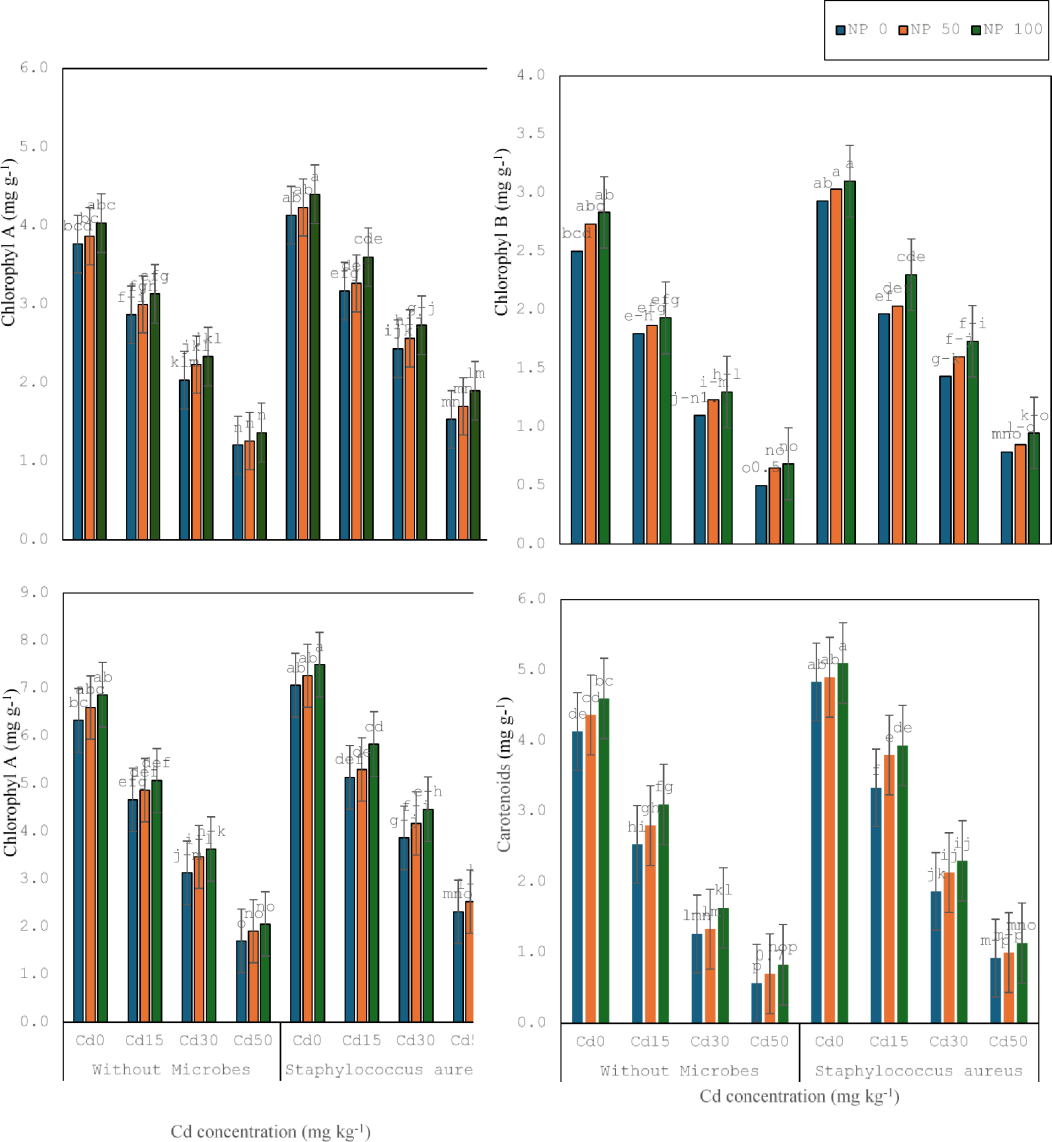
Effect of microbes on chlorophyll a, b and total chlorophyll and carotenoids of wheat plant under the cadmium (0, 15, 30 and 50 mg kg^− 1^). Showing standard deviation at *p* ≤ 0.05 level with mean of three replications.

The current research was conducted to estimate the bio-toxic impacts of cadmium on several structural, physical, and biochemical constraints of wheat and to evaluate the reducing ability of cadmium opposing bacteria (*S. aureus* M1) in a synergistic relationship with TiO_2_ NPs as shown in (Fig. 2). It was noticed that Cd 15 mg kg^− 1^ decreased the total chlorophyll, a, b, and carotenoids (26, 28, 24, and 39%) compared to the control. It was noticed that Cd 30 mg kg^− 1^ decreased the total chlorophyll, a, b, and carotenoids (32, 29,38, and 50%) compared to Cd 15 mg kg^− 1^. It was noticed that Cd 50 mg kg^− 1^ decreased the total chlorophyll, a, b, and carotenoids (45, 40, 54, and 55%) related to Cd 30 mg kg^− 1^.

*S. aureus* M1 inoculation boosted the total chlorophyll, a, b, and carotenoids (12, 9, 17, and 17%) under no toxicity, compared without inoculation. *S. aureus* M1 inoculation enhanced the total chlorophyll, a, b, and carotenoids (10, 8, 9, and 31%) under Cd 15 mg kg^− 1^, associated without inoculation. *S. aureus* M1 inoculation improved the total chlorophyll, a, b, and carotenoids (23, 19, 30, and 47%) under Cd 30 mg kg^− 1^, related without inoculation. Similarly, *S. aureus* M1 inoculation augments the total chlorophyll, a, b, and carotenoids (23, 19, 30, and 47%) under Cd 50 mg kg^− 1^, linked without inoculation.

TiO_2_ 50mg L^− 1^ NPs enhanced the total chlorophyll, a, b, and carotenoids (4, 3, 9, and 6%) under no toxicity, contrasted to control without TiO_2_ 50mg L^− 1^ NPs. Although TiO_2_ 50mg L^− 1^ NPs enhanced the total chlorophyll, a, b, and carotenoids (4, 5, 4, and 10%) under Cd 15 mg kg^− 1^, associated without TiO_2_ 50mg L^− 1^ NPs. TiO_2_ 50mg L^− 1^ NPs enhanced the total chlorophyll, a, b, and carotenoids (10, 9, 12, and 5%) under Cd 30 mg kg^− 1^, related without TiO_2_ 50mg L^− 1^ NPs. TiO_2_ 50mg L^− 1^ NPs enhanced the total chlorophyll, a, b, and carotenoids (12, 4, 30, and 24%) under Cd 50 mg kg^− 1^, contrary without TiO_2_ 50mg L^− 1^ NPs. Similarly, TiO_2_ 100mg L^− 1^ NPs enhanced the total chlorophyll, a, b, and carotenoids (4, 3, 5, and 11%) under Cd 15 mg kg^− 1^, parallel to TiO_2_ 50mg L^− 1^ NPs. TiO_2_ 100mg L^− 1^ NPs greater the total chlorophyll, a, b, and carotenoids (3, 4, 5, and 22%) under Cd 30 mg kg^− 1^, contrary to TiO_2_ 50mg L^− 1^ NPs. Correspondingly, TiO_2_ 100mg L^− 1^ NPs enhanced the total chlorophyll, a, b, and carotenoids (7, 8, 5, and 18%) under Cd 50 mg kg^− 1^, contrasted to TiO_2_ 50mg L^− 1^ NPs.

Co-application of TiO_2_ 50mg L^− 1^ NPs and *S. aureus* M1 inoculation augmented the total chlorophyll, a, b, and carotenoids (3, 2, 3, and 2%) under no stress, parallel with respective treatment without TiO_2_ 50mg L^− 1^ NPs. The co-application of TiO_2_ 50mg L^− 1^ NPs and *S. aureus* M1 inoculation enhanced the total chlorophyll, a, b, and carotenoids (2, 3, 3, and 14%) and carotenoid (14%) under Cd 15 mg kg^− 1^, associated with corresponding treatment without TiO_2_ 50mg L^− 1^ NPs. The co-application of TiO_2_ 50mg L^− 1^ NPs and *S. aureus* M1 inoculation enhanced the total chlorophyll, a, b, and carotenoids (8, 5, 12, and 14%) under Cd 30 mg kg^− 1^, linked with corresponding treatment without TiO_2_ 50mg L^− 1^ NPs. The co-application of TiO_2_ 50mg L^− 1^ NPs and *S. aureus* M1 inoculation enhanced the total chlorophyll, a, b, and carotenoids (9, 11, 8, and 8%) under Cd 50 mg kg^− 1^, contrary with corresponding treatment without TiO_2_ 100mg L^− 1^ NPs. Co-application of TiO_2_ 100mg L^− 1^ NPs and *S. aureus* M1 inoculation enhanced the total chlorophyll, a, b, and carotenoids (3, 4, 2, and 4%) under no stress, contrasted with respective treatment without TiO_2_ 100mg L^− 1^ NPs. The co-application of TiO_2_ 100mg L^− 1^ NPs and *S. aureus* M1 inoculation enhanced the total chlorophyll, a, b, and carotenoids (10, 10, 9, and 4%) under Cd 15 mg kg^− 1^, in comparison with corresponding treatment without TiO_2_ 100mg L^− 1^ NPs. The co-application of TiO_2_ 100mg L^− 1^ NPs and *S. aureus* M1 inoculation enhanced the total chlorophyll, a, b, and carotenoids (7, 6, 8, and 9%) under Cd 30 mg kg^− 1^, contrary with following treatment without TiO_2_ 100mg L^− 1^ NPs. The co-application of TiO_2_ 100mg L^− 1^ NPs and *S. aureus* M1 inoculation enhanced the total chlorophyll, a, b, and carotenoids (12, 10, 11, and 13%) following Cd 50 mg kg^− 1^, equated with corresponding treatment without TiO_2_ 100mg L^− 1^ NPs.

### Evaluation of EL, H2O2 and MDA

**Fig. 3:**
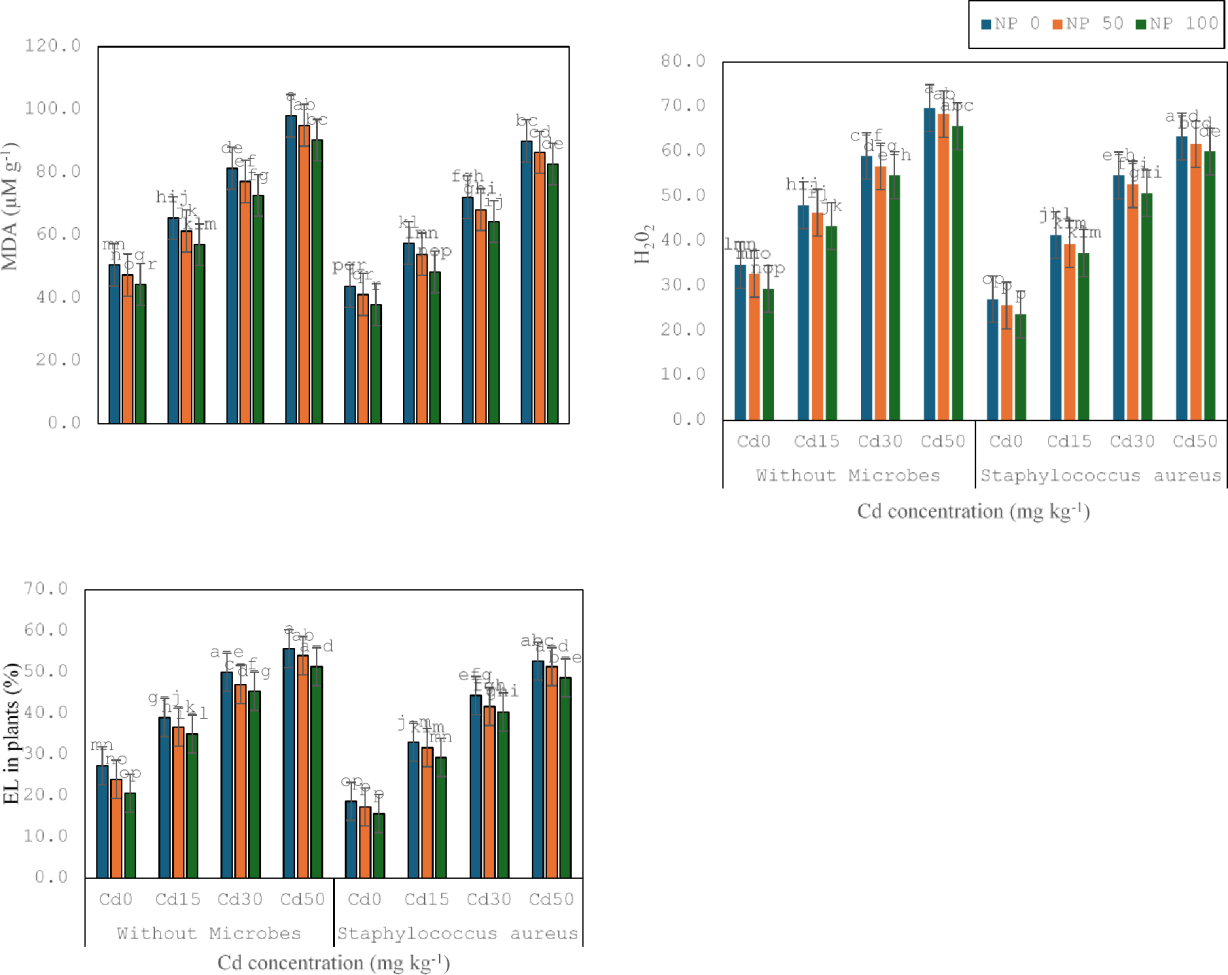
Effect of microbes on MDA, H_2_O_2_ and EL of wheat plant under the cadmium (0, 15, 30 and 50 mg kg^− 1^). Showing standard deviation at *p* ≤ 0.05 level with mean of three replications.

In this section, the bio-toxic consequences of cadmium on oxidative stress markers were evaluated for the increasing potential of cadmium-opposing bacterial strains (*S. aureus* M1) in a synergistic relationship with TiO_2_ NPs. It was noticed that Cd stress increased electrolyte leakage (EL), hydrogen peroxide (H_2_O_2_), and malondialdehyde (MDA). (Fig. 3). It was noticed that Cd 15 mg kg^− 1^ decreased MDA (29%), H_2_O_2_ (38%), and EL (43%) as related to control. It was noticed that Cd 30 mg kg^− 1^ decreased MDA (24%), H_2_O_2_ (23%), and EL (28%) as contrasted to Cd 15 mg kg^− 1^. It was noticed that Cd 50 mg kg^− 1^ reduced MDA (20%), H_2_O_2_ (18%), and EL (111%) as contrasted to Cd 30 mg kg^− 1^. *S. aureus* M1 inoculation enhanced MDA (13), H_2_O_2_ (22%), and EL (31%) under no stress, compared to without inoculation. Whereas, with *S. aureus* M1 inoculation enhanced the MDA (13%), H_2_O_2_ (14), and EL (15%) under Cd 15 mg kg^− 1^, contrasted without inoculation. *S. aureus* M1 inoculation enhanced the MDA (11%), H_2_O_2_ (45%), and EL (11%) under Cd 30 mg kg^− 1^, related without inoculation. Similarly, with *S. aureus* M1 inoculation enhanced MDA (8%), H_2_O_2_ (9%), and EL (5%) under Cd 50 mg kg^− 1^, in comparison without inoculation. TiO_2_ 50mg L^− 1^ NPs reduced the MDA (6%), H_2_O_2_ (6%), and EL (12%) under no stress, associated with control without TiO_2_ 50mg L^− 1^ NPs. While TiO_2_ 50mg L^− 1^ NPs decreased MDA (6%), H_2_O_2_ (3%), and EL (6%) under Cd 15 mg kg^− 1^, contrary without TiO_2_ 50mg L^− 1^ NPs.

TiO_2_ 50mg L^− 1^ NPs reduced the MDA (5%), H_2_O_2_ (4%), and EL (6%) following Cd 30 mg kg^− 1^, parallel to no application of TiO_2_ 50mg L^− 1^ NPs. TiO_2_ 50mg L^− 1^ NPs reduced the MDA (3%), H_2_O_2_ (2%), and EL (3%) following Cd 50 mg kg^− 1^, equated to no application of TiO_2_ 50mg L^− 1^ NPs. Similarly, TiO_2_ 100mg L^− 1^ NPs enhanced MDA (7%), H_2_O_2_ (6%) and EL (5%) under Cd 15 mg kg^− 1^, contrasted to TiO_2_ 50mg L^− 1^ NPs. TiO_2_ 100mg L^− 1^ NPs reduced the MDA (6%), H_2_O_2_ (4%), and EL (4%) under Cd 30 mg kg^− 1^, in comparison to TiO_2_ 50mg L^− 1^ NPs. Correspondingly, TiO_2_ 100mg L^− 1^ NPs decreased MDA (5%), H_2_O_2_ (4%) and EL (5%) under Cd 50 mg kg^− 1^, contrary to TiO_2_ 50mg L^− 1^ NPs co-application of TiO_2_ 50mg L^− 1^ NPs and *S. aureus* M1 inoculation decreased the MDA (6%), H_2_O_2_ (5%) and EL (7%) under no stress, related with following treatment without TiO_2_ 50mg L^− 1^ NPs. The co-application of TiO_2_ 50mg L^− 1^ NPs and *S. aureus* M1 inoculation decreased MDA (6%), H_2_O_2_ (15%), and EL (4%) under Cd 15 mg kg^− 1^, linked with corresponding treatment without TiO_2_ 50mg L^− 1^ NPs. The co-application of TiO_2_ 50mg L^− 1^ NPs and *S. aureus* M1 inoculation decreased the MDA (5%), H_2_O_2_ (4%) and EL (6%) under Cd 30 mg kg^− 1^, rivalled with corresponding treatment without TiO_2_ 50mg L^− 1^ NPs.

The co-application of TiO_2_ 50mg L^− 1^ NPs and *S. aureus* M1 inoculation decreased the MDA (4%), H_2_O_2_ (3%), and EL (2%) following Cd 50 mg kg^− 1^, contrary to corresponding treatment without TiO_2_ 100mg L^− 1^ NPs. Co-application of TiO_2_ 100mg L^− 1^ NPs and *S. aureus* M1 inoculation enhanced the MDA (7%), H_2_O_2_ (8%), and EL (10%) under no stress, in comparison with respective treatment without TiO_2_ 100mg L^− 1^ NPs. The co-application of TiO_2_ 100mg L^− 1^ NPs and *S. aureus* M1 inoculation decreased the MDA (10%), H_2_O_2_ (5%), and EL (7%) under Cd 15 mg kg^− 1^, associated with corresponding treatment without TiO_2_ 100mg L^− 1^ NPs. The co-application of TiO_2_ 100mg L^− 1^ NPs and *S. aureus* M1 inoculation decreased the MDA (11%), H_2_O_2_ (4%), and EL (9%) under Cd 30 mg kg^− 1^, parallel with corresponding treatment without TiO_2_ 100mg L^− 1^ NPs. The co-application of TiO_2_ 100mg L^− 1^ NPs and *S. aureus* M1 inoculation enhanced the MDA (4%), H_2_O_2_ (3%), and EL (5%) following Cd 50 mg kg^− 1^, equated with corresponding treatment without TiO_2_ 100mg L^− 1^ NPs.

### Antioxidant enzyme activities

**Fig. 4:**
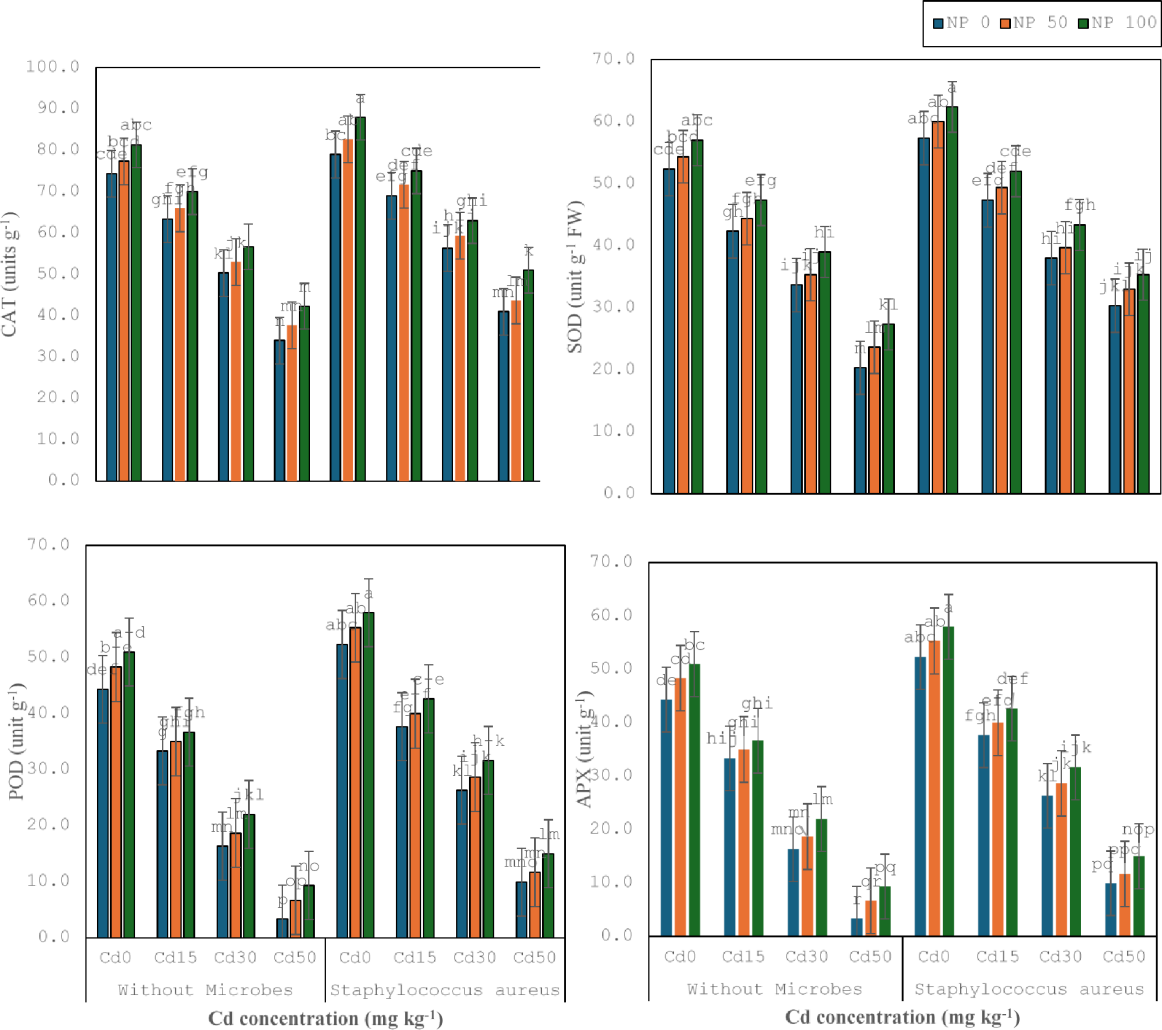
Effect of microbes on CAT, SOD and POD of wheat plant under the cadmium (0, 15, 30 and 50 mg kg^− 1^). Showing standard deviation at *p* ≤ 0.05 level with mean of three replications.

The existing study was carried out to estimate the bio-toxic impacts of cadmium on antioxidative enzymes to evaluate the reducing ability of cadmium-opposing bacterial strains (*S. aureus* M1) in synergistic relationships with TiO_2_ NPs. It was noticed that Cd stress decreased CAT, POD, POD, and APX (Fig. 4). It was noticed that Cd 15 mg kg^− 1^ diminished CAT (15%), SOD (19%), POD (19%) and APX (25%) as compared to control. It was noticed that Cd 30 mg kg^− 1^ lessened CAT (21%), SOD (20%), POD (31%), and APX (51%) as contrasted to Cd 15 mg kg^− 1^. It was noticed that Cd 50 mg kg^− 1^ declined CAT (32%), SOD (39%), POD (40%), and APX (80%) as contrary to Cd 30 mg kg^− 1^. *S. aureus* M1 inoculation enhanced CAT (6%), SOD (10%), POD (12%) and APX (18%) under no stress, compared without inoculation. Whereas *S. aureus* M1 inoculation enhanced the CAT (8%), SOD (12%), POD (12%), and APX (13%) under Cd 15 mg kg^− 1^, compared without inoculation. *S. aureus* M1 inoculation enhanced the CAT (12%), SOD (12%), POD (22%), and APX (61%) under Cd 30 mg kg^− 1^, in comparison without inoculation. Similarly, with *S. aureus* M1 inoculation enhanced CAT (21%), SOD (49%), POD (56%), and APX (198%) under Cd 50 mg kg^− 1^, in comparison without inoculation.

TiO_2_ 50mg L^− 1^ NPs upsurge the CAT (4%), SOD (3%), POD (5%), and APX (6%) under no toxicity, in comparison to control without TiO_2_ 50mg L^− 1^ NPs. While TiO_2_ 50mg L^− 1^ NPs enhanced CAT (4%), SOD (5%), POD (3%), and APX (5%) under Cd 15 mg kg^− 1^, contrasted without TiO_2_ 50mg L^− 1^ NPs. TiO_2_ 50mg L^− 1^ NPs improved the CAT (5%), SOD (5%), POD (9%), and APX (81%) under Cd 30 mg kg^− 1^, contrary without TiO_2_ 50mg L^− 1^ NPs. TiO_2_ 50mg L^− 1^ NPs momentous upsurge the CAT (11%), SOD (16%), POD (24%), and APX (100%) under Cd 50 mg kg^− 1^, in comparison without TiO_2_ 50mg L^− 1^ NPs. Similarly, TiO_2_ 100mg L^− 1^ NPs enhanced CAT (6%), SOD (7%), POD (8%) and APX (5%) under Cd 15 mg kg^− 1^, contrasted to TiO_2_ 50mg L^− 1^ NPs. TiO_2_ 100mg L^− 1^ NPs augmented the CAT (7%), SOD (10%), POD (14%), and APX (18%) under Cd 30 mg kg^− 1^, contrary to TiO_2_ 50mg L^− 1^ NPs. Correspondingly, TiO_2_ 100mg L^− 1^ NPs enhanced CAT (12%), SOD (15%), POD (10%) and APX (40%) under Cd 50 mg kg^− 1^, contrasted to TiO_2_ 50mg L^− 1^ NPs. Co-application of TiO_2_ 50mg L^− 1^ NPs and *S. aureus* M1 inoculation enhanced the CAT (5%), SOD (5%), POD (4%), and APX (6%) under no stress, in comparison with respective treatment without TiO_2_ 50mg L^− 1^ NPs. The co-application of TiO_2_ 50mg L^− 1^ NPs and *S. aureus* M1 inoculation enhanced CAT (4%), SOD (4%), POD (5%) and APX (6%) under Cd 15 mg kg^− 1^, contrary with corresponding treatment without TiO_2_ 50mg L^− 1^ NPs.

The co-application of TiO_2_ 50mg L^− 1^ NPs and *S. aureus* M1 inoculation enhanced the CAT (5%), SOD (4%), POD (6%), and APX (9%) under Cd 30 mg kg^− 1^, in comparison with corresponding treatment without TiO_2_ 50mg L^− 1^ NPs. The co-application of TiO_2_ 50mg L^− 1^ NPs and *S. aureus* M1 inoculation enhanced the CAT (7%), SOD (9%), POD (5%), and APX (17%) under Cd 50 mg kg^− 1^, contrasted with corresponding treatment without TiO_2_ 100mg L^− 1^ NPs. Co-application of TiO_2_ 100mg L^− 1^ NPs and *S. aureus* M1 inoculation enhanced the CAT (6%), SOD (4%), POD (5%), and APX (5%) under no stress, compared with corresponding treatment without TiO_2_ 100mg L^− 1^ NPs. The co-application of TiO_2_ 100mg L^− 1^ NPs and *S. aureus* M1 inoculation enhanced the CAT (5%), SOD (5%), POD (6%), and APX (7%) under Cd 15 mg kg^− 1^, contrasted with corresponding treatment without TiO_2_ 100mg L^− 1^ NPs. The co-application of TiO_2_ 100mg L^− 1^ NPs and *S. aureus* M1 inoculation enhanced the CAT (6%), SOD (10%), POD (7%), and APX (10%) under Cd 30 mg kg^− 1^, in comparison with corresponding treatment without TiO_2_ 100mg L^− 1^ NPs. The co-application of TiO_2_ 100mg L^− 1^ NPs and *S. aureus* M1 inoculation enhanced the CAT (17%), SOD (7%), POD (8%), and APX (29%) under Cd 50 mg kg^− 1^, contrasted with corresponding treatment without TiO_2_ 100mg L^− 1^ NPs.

### Accumulation of metals

**Fig. 5:**
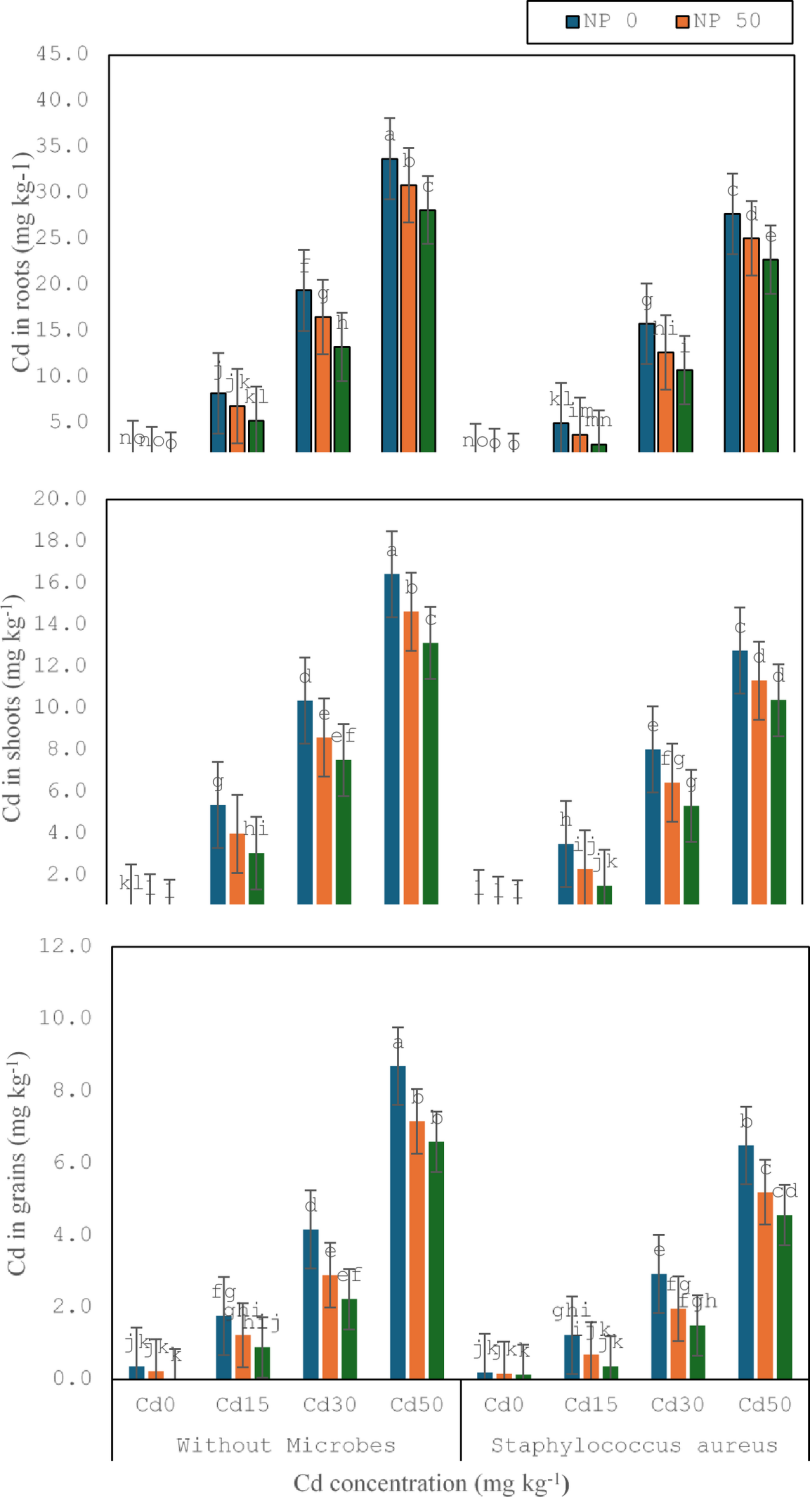
Effect of microbes on Cd in roots, Cd in shoots and Cd in grains of wheat plant under the cadmium (0, 15, 30 and 50 mg kg^− 1^). Showing standard deviation at *p* ≤ 0.05 level with mean of three replications.

Cd uptake was significantly increased under Cd (15, 30, and 50 mg kg^− 1^) toxicity as contrasted to control, as shown in (Fig. 5). TiO_2_ 50mg L^− 1^ NPs implication significantly lessened the Cd absorption and accumulation in roots (33%), shoots (57%), and grains (36%) without any stress compared to the respective treatment. It was noticed that TiO_2_ 50mg L^− 1^ NPs employment significantly declined the Cd concentration in roots (7%), shoots (11%), and grains (9%) under Cd 15 mg kg^− 1^ contrasted to the corresponding treatment. TiO_2_ 50mg L^− 1^ NPs employment significantly abridged the Cd concentration in roots (6%), shoots (9%), and grains (10%) under Cd 30 mg kg^− 1^ in comparison to the corresponding treatment. TiO_2_ 50mg L^− 1^ NPs application significantly attenuates the Cd concentration in roots (5%), shoots (7%), and grains (18%) under Cd 50 mg kg^− 1^ contrary to the corresponding treatment. Similarly, TiO_2_ 100 mg L^− 1^ NPs implication pointedly abridged the Cd concentration in roots (24%), shoots (20%), and grains (26%) under Cd 15 mg kg^− 1^ contrasted to the corresponding treatment. TiO_2_ 100 mg L^− 1^ NPs application significantly reduced the Cd concentration in roots (10%), shoots (15%), and grains (19%) under Cd 30 mg kg^− 1^ contrary to the corresponding treatment. TiO_2_ 100mg L^− 1^ NPs employment significantly abridged the Cd concentration in roots (12%), shoots (14%), and grains (17%) under Cd 50 mg kg^− 1^ in comparison to corresponding treatment.

*S. aureus* M1 inoculation inhibited Cd uptake in roots (39%), shoots (23%), and grains (41%) under Cd 15 mg kg^− 1^ toxicity, in comparison without inoculation. *S. aureus* M1 inoculation inhibited Cd uptake in roots (26%), shoots (21%), and grains (19%) under Cd 30 mg kg^− 1^ toxicity, contrasted without inoculation. *S. aureus* M1 inoculation inhibited Cd uptake in roots (13%), shoots (7%), and grains (9%) under Cd 50 mg kg^− 1^ toxicity, contrary without inoculation. *S. aureus* M1 inoculation and TiO_2_ 50mg L^− 1^ NPs inhibited Cd absorption in roots (31%), shoots (22%), and grains (17%) under Cd 15 mg kg^− 1^ toxicity, contrasted without inoculation. *S. aureus* M1 inoculation and TiO_2_ 50mg L^− 1^ NPs inhibited Cd absorption in roots (46%), shoots (37%), and grains (38%) under Cd 30 mg kg^− 1^ toxicity, contrasted without inoculation. *S. aureus* M1 inoculation and TiO_2_ 50mg L^− 1^ NPs inhibited Cd absorption in roots (23%), shoots (29%), and grains (24%) under Cd 50 mg kg^− 1^ toxicity, contrary without inoculation. Similarly, *S. aureus* M1 inoculation and TiO_2_ 100mg L^− 1^ NPs inhibited Cd absorption in roots (38%), shoots (41%), and grains (34%) under Cd 15 mg kg^− 1^ toxicity, contrary without inoculation. *S. aureus* M1 inoculation and TiO_2_ 100mg L^− 1^ NPs inhibited Cd absorption in roots (12%), shoots (21%), and grains (19%) under Cd 30 mg kg^− 1^ toxicity, in comparison without inoculation. *S. aureus* M1 inoculation and TiO_2_ 100mg L^− 1^ NPs inhibited Cd absorption in roots (34%), shoots (23%), and grains (27%) under Cd 50 mg kg^− 1^ toxicity, contrasted without inoculation.

## Discussion

The study shows that nanoparticles added to the earth have modified the structure of the community of prokaryotes except for fungus, in fact they boost the energy of good bacteria and stimulate the growth of plants. Similarly, nanoparticles and microbes alleviated the toxicity of SDZ, Cd and Cr in spinach and increased growth and yield^31–35^.

Particular heavy metal-resistant plant growth-promoting rhizobacteria (PGPRs) have been found to possess additional mechanisms that mitigate metal uptake or translocation within plants. These mechanisms involve reducing metal bioavailability in the soil through various processes, including bioaccumulation, biosorption, precipitation, biotransformation (via methylation, demethylation, volatilization, complex formation, oxidation, or reduction), complexation, and alkalization, ultimately minimizing metal toxicity and promoting plant health^36^. Plants thriving in heavy metal-contaminated soils often harbor diverse microorganisms that confer beneficial effects, including enhanced plant growth and augmented tolerance to metal-induced stress, promoting plant resilience in polluted environments^37^. Chromium-reducing bacteria possess a unique capacity to mitigate Cr toxicity through a transformative process. By leveraging bioaccumulation and biosorption mechanisms, these bacteria effectively convert the highly toxic Cr^6+^ into the less harmful Cr^3+^ within the rhizosphere, promoting a safer and more sustainable environment^38,39^.

A compensatory response is triggered once biophysical barriers are compromised, allowing heavy metals to infiltrate plant cells. Plants respond by accumulating protective metabolites and activating their cellular defense systems, thereby mitigating the deleterious effects of reactive oxygen species (ROS) generated by heavy metal exposure^40^. Our study depicted that Cd spiking (15-30 mg kg^− 1^) significantly affects the wheat plant growth attributes such as root and shoot lengths, root and shoot fresh and dry weights, no. of tillers, and grain weight, which is similar to a former study finding that the toxicity of cadmium reduced the growth attributes such as biomass (fresh and dry), grain weight, tiller plant^− 1^ and grain production plant^− 1^^41^. While inoculation with strains *Staphylococcus Aureus* improved the physiological characteristics of wheat plants under Cd toxicity, which is consistent with earlier study outcomes that application of metal tolerant plant growth promoting bacteria (*Cellulosimicrobium* sp) exhibit high heavy metal resistance and enhanced the growth parameters of *Medicago sativa* (Alfalfa) plants under Cr stress^42^. Likewise, foliar application of TiO_2_ NPs (50, 100 mg kg^− 1^) augmented the physiological attributes of wheat plants under Cd stress. A previous study showed that foliar spray of several levels of TiO_2_ NPs (20, 40, 60, 80mg L^− 1^) increased the physiological attributes in wheat plants under salinity stress^43,44^. Besides this, the collective application of TiO_2_ NPs and microbial strains of *Staphylococcus Aureus* significantly boosted the growth attributes of wheat under Cd toxicity. Similar results showed in former studies that joint employment of strains *Staphylococcus aureus* K1 and ZnO nanoparticles (0, 50, 100 mg/L) increased the wheat plant growth and physiological characteristics^45^.

Existing studies demonstrated that Cd stress significantly affects the wheat plant chlorophyll contents such as chlorophyll a, b, total chlorophyll, and carotenoids. This is similar to a former study finding that cadmium toxicity suppressed photosynthetic pigments across different plant species and reduced chlorophyll, possibly caused by substituting chlorophyll structure, consequently causing massive uptake of Cd^46^. While inoculation with strains *Staphylococcus Aureus* improved the chlorophyll contents of wheat plants under Cd toxicity, which is consistent with earlier study outcomes that employment of *Bacillus* strains regulates Cd^2+^ biosorption mechanisms and improves rice seedlings growth, chlorophyll contents^47^. Likewise, foliar application of TiO_2_ NPs (50, 100 mg kg^− 1^) augmented the chlorophyll contents of wheat plants under Cd stress. A previous study exhibited similar results that the application of anatase nano-TiO_2_ of two diameters (50–68 nm) showed positive effects on chlorophyll a, b and total chlorophyll and nutritional quality of okra (*Abelmoschus esculentus*) plants^48^. Besides this, the collective application of TiO_2_ NPs and microbial strains *Staphylococcus Aureus* significantly boosted the chlorophyll contents of wheat under Cd toxicity. Similar results showed in former studies that combined application of *B. pumilus* and ZnO NPs (20ppm), and TiO_2_ NPs (10ppm) increased the growth and photosynthetic pigments (Carotenoids, chlorophyll a, b) in maize plants under Cd (20ppm) toxicity^49^.

The current study elaborated that Cd stress significantly affects the wheat plants activities of antioxidants like SOD, POD, APX, and CAT and oxidants including EL, MDA, and H_2_O_2_. Similar to a former study finding that the toxicity of Cd (50 mg kg^− 1^) abridged the antioxidant enzymes activities like SOD, POD, CAT, and APX and amplified the oxidative stress markers such as MDA, H_2_O_2_, and OH^−^ in wheat^50^. While inoculation with strains *Staphylococcus Aureus* improved the activities of antioxidant and oxidant of wheat plants under Cd toxicity, which is consistent with earlier study outcomes that application of *S. aureus* K1 regulated the wheat plants’ growth and antioxidant enzymatic activities by reducing oxidative stress and Cr toxicity through conversion of Cr^6+^ to Cr^3+^^23^. Likewise, foliar application of TiO_2_ NPs (50, 100 mg kg^− 1^) augmented wheat plants’ antioxidant and oxidant activities under Cd stress. A previous study exhibited similar results that the application of TiO_2_-NPs upgraded antioxidant enzyme activities like guaiacol peroxidase (GPX), ascorbate peroxidase (APX), catalase (CAT) and superoxide dismutase (SOD) in *Spirodela polyrrhiza* plants and lessens biosynthesis of malondialdehyde (MDA) and hydrogen peroxide (H_2_O_2_)^51^.

Besides this, the collective application of TiO_2_ NPs and microbial strains of *Staphylococcus Aureus* significantly boosted the activities of antioxidant and oxidant of wheat under Cd toxicity. Similar results showed in former studies that combined application of Fe nanoparticles and Chromium-resistant bacterium *Staphylococcus aureus* reduced the surplus of electrolyte leakage, hydrogen peroxide, and malondialdehyde and enhanced the defense system of rice plants by increasing antioxidants enzyme activities under Cr toxicity^52^. Our study depicted that Cd stress significantly amplified the Cd accumulation in wheat plants’ roots, shoots, and grains. Similar to a former research finding, the toxicity of Cd (20 mg kg^− 1^) by applying CdCl_2_ in soil enhanced the Cd concentration in roots and shoots in wheat plants^53^. In comparison, inoculation with strains of *Staphylococcus Aureus* alleviates the Cd concentration in wheat plants’ roots, shoots, and grains. Consistent with earlier study outcomes, the application of Cd-resistant strain WRS8 abridged the wheat tissue Cd translocation by growing root surface Cd adsorption and declining wheat root Cd absorption and transport-related gene expression^54^. Likewise, foliar application of TiO_2_ NPs (50, 100 mg kg^− 1^) augmented the physiological attributes of wheat plants under Cd stress. A previous study showed that the impact of TiO_2_ NPs (0, 100, 250mg L^− 1^) significantly reduced the Cd level in roots and shoots in maize^55^. Besides this, the collective application of TiO_2_ NPs and microbial strains of *Staphylococcus Aureus* significantly declined the Cd concentration in wheat roots, shoots, and grains. Similarly, former studies showed that collective employment of PGPR and Fe foliar implications mitigate Cr toxicity in maize plants^56^.

## Conclusions

The current study explored that cadmium toxicity pointedly decreased wheat plant growth and yield characteristics, antioxidant enzyme activities, and Cd accumulation in shoots and grains. Besides this, the combined application of TiO_2_ NPs and *Staphylococcus Aureus* upsurge the plant’s physiological attributes and yield. Similarly, the collective application significantly attenuates oxidative stress (MDA, H_2_O_2,_ and EL) by increasing antioxidant enzyme activities (SOD, POD, APX, and CAT). Moreover, applying TiO_2_ NPs and *Staphylococcus Aureus* reduced the Cd concentration in roots, shoots, and grains. Overall, this research suggested that TiO_2_ NPs and *Staphylococcus Aureus*, in combination, are efficacious applications for Cd alleviation and improving wheat yield. Integrating Cd-resistant microbes and TiO_2_ nanoparticles in agricultural practices offers a groundbreaking, eco-friendly solution for the remediation of cadmium-contaminated soils. By adopting this innovative technology, farmers can ensure a safer and more sustainable food production process, minimizing the risks associated with cadmium pollution. This eco-friendly biotechnology solution must be scaled up and implemented extensively. Policymakers and stakeholders must prioritize adopting this innovative technology to remediate heavy metal-contaminated soils, promoting environmental sustainability and public health. Conducting in-depth field research is crucial to guarantee future food security and sustainable agriculture. Furthermore, investigations should also focus on diverse soil types and varied crop cultivars to develop tailored solutions, ultimately enhancing crop resilience, productivity, and environmental stewardship.

### Environment implication

Cadmium (Cd) is a toxic heavy metal that can accumulate in wheat, posing a significant threat to human health and the environment. The bio-toxic effects of Cd can be alleviated using Cd-resistance microbes, such as Staphylococcus aureus and TiO_2_ nanoparticles. The use of Cd-resistance microbes, such as Staphylococcus aureus, and TiO_2_ nanoparticles can alleviate the bio-toxic effects of Cd in wheat. Cd-resistance microbes can improve soil health by promoting the growth of beneficial microorganisms. These technologies can promote sustainable agriculture by reducing the need for chemical fertilizers and pesticides. These technologies have significant environmental implications, including reduced Cd accumulation, improved soil health, and promotion of sustainable agriculture.

## Data Availability

All data generated or analyzed during the study are included in this article.
